# Investigation on Phenomics of Traditional Chinese Medicine from the Diabetes

**DOI:** 10.1007/s43657-023-00146-6

**Published:** 2024-09-02

**Authors:** Boxun Zhang, Lijuan Zhou, Keyu Chen, Xinyi Fang, Qingwei Li, Zezheng Gao, Fengmei Lian, Min Li, Jiaxing Tian, Linhua Zhao, Xiaolin Tong

**Affiliations:** 1 Institute of Metabolic Diseases, Guang’anmen Hospital, China Academy of Chinese Medical Sciences, Beijing, 100053 China; 2 Graduate College, Beijing University of Chinese Medicine, Beijing, 100029 China; 3 Northeast Asia Research Institute of Traditional Chinese Medicine, Changchun University of Chinese Medicine, Changchun, 130117 China

**Keywords:** Traditional Chinese medicine, Phenomics, Diabetes, State-target theory, Integration of Chinese and Western medicine

## Abstract

With thousands of years of application history, traditional Chinese medicine (TCM) has unique advantages in the prevention of various chronic diseases, and in recent years, the development of TCM has presented a situation where opportunities and challenges coexist. Phenomics is an emerging area of life science research, which has numerous similarities to the cognitive perspective of TCM. Thus, how to carry out the interdisciplinary research between TCM and phenomics deserves in-depth discussion. Diabetes is one of the most common chronic non-communicable diseases around the world, and TCM plays an important role in all stages of diabetes treatment, but the molecular mechanisms are difficult to elucidate. Phenomics research can not only reveal the hidden scientific connotations of TCM, but also provide a bridge for the confluence and complementary between TCM and Western medicine. Facing the challenges of the TCM phenomics research, we suggest applying the State-target theory (STT) to overall plan relevant researches, namely, focusing on the disease development, change trends, and core targets of each stage, and to deepen the understanding of TCM disease phenotypes and the therapeutic mechanisms of herbal medicine.

**Supplementary Information:**

The online version contains supplementary material available at 10.1007/s43657-023-00146-6.

## Introduction

Traditional Chinese medicine (TCM) is a valuable asset of the Chinese nation and plays an important role in the prevention and treatment of multiple major chronic diseases (Zhang et al. 2021), while the pharmacodynamic material basis and the target mechanism need to be further clarified, and the interdisciplinary innovation provides a good opportunity for the development of TCM. After the upsurge of the human genome research, phenomics has risen rapidly and has achieved fruitful outcomes in recent years. Phenome refers to a series of measurable characteristics generated through the complex interaction among genes, epigenetics, symbiotic microorganisms, diet and environmental exposure, including physical, chemical, and biological characteristics in individuals and populations; phenomics, first proposed by professor S. Garan of the University of California in 1996, is a subject that studies all phenotypes from micro- to macro-level, covering transcription, protein, metabolism, cells, organs, psychology, and other aspects (Jin 2022). In thousands of years of clinical practice, a large number of TCM practitioners have comprehensively observed and recorded various types of phenotypic information about human psychology, behavior, physiology, pathology, and drug reactions. By making full use of applying genomics, transcriptomics, proteomics, metabolomics, and microbiomics, phenomics studies in TCM can deepen the cognitive level of the human physiology and pathology, explain the scientific connotation of TCM syndromes, reveal the effective material basis and functional mechanism of herbs, so as to reveal the essence of the life process more systematically and completely, and eventually, promote the integration of Chinese and Western medicine (Houle et al. 2010; Cheng et al. 2016; Yuan et al. 2022). Diabetes is a major chronic disease that poses a huge health threat to people all over the world, and a series of TCM treatments have been proved to be effective in preventing and treating the disease. Taking diabetes as an example, this review will discuss how to use phenomics to open the “black box” of TCM, and promote the innovative development of TCM.

## Phenomics and the Construction of a Modern TCM Diagnosis and Treatment System of the Diabetes

**Fig. 1:**
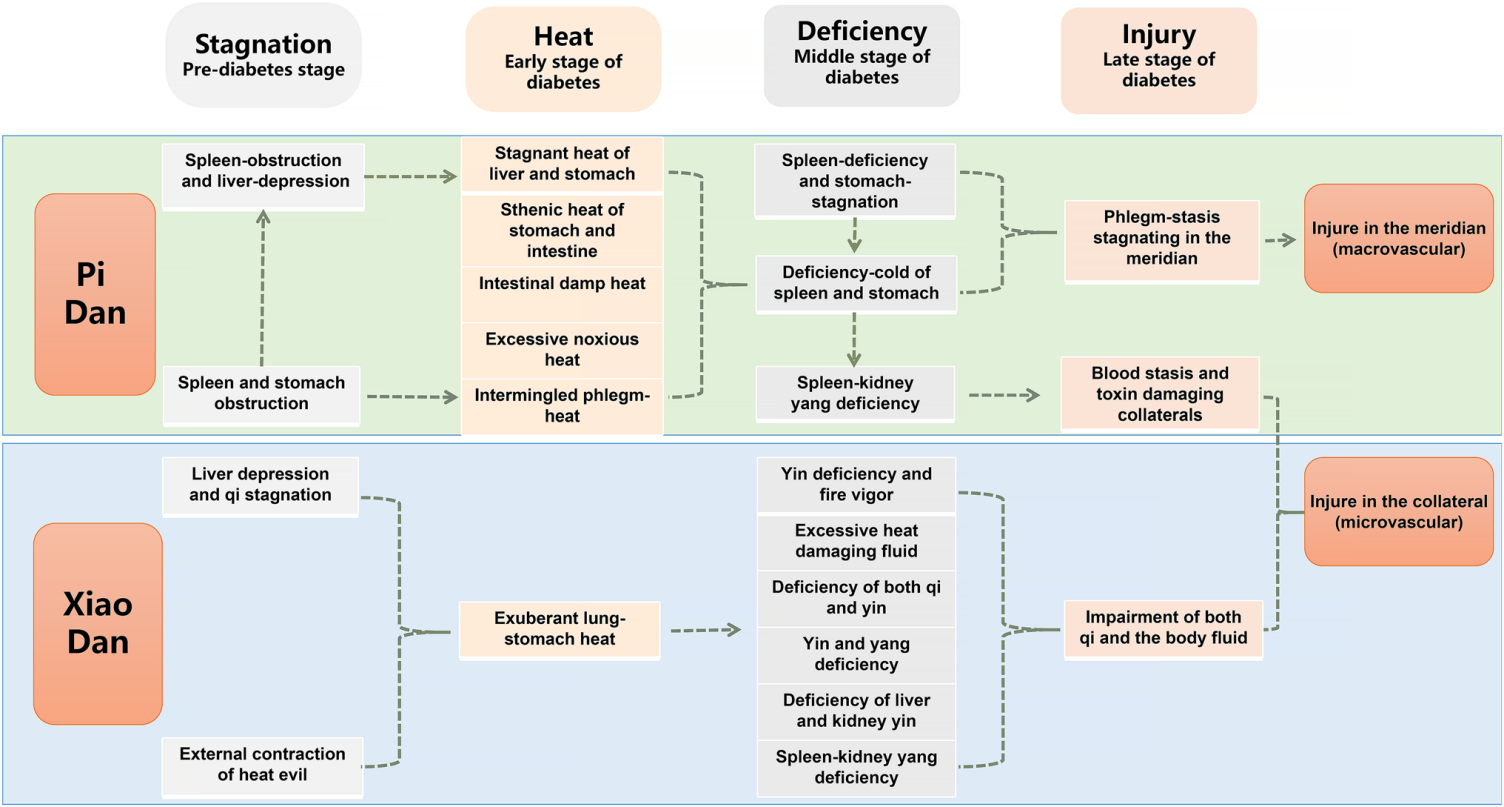
TCM diagnosis and treatment system of the diabetes—“classification, staging and syndrome differentiation strategy”

Diabetes belongs to the category of “Consumptive Thirst disease” in TCM, but due to the limitations of the times, the traditional “Consumptive Thirst” theory could not be effectively adapted to the modern clinical practice. Based on a large number of clinical practices and a series of evidence-based studies, our team proposed the modern TCM diagnosis and treatment system. The “classification, staging and syndrome differentiation strategy” is the core of the new system, and specifically, we classified diabetes into two main types: Pi Dan and Xiao Dan; meanwhile, we considered that the development of diabetes usually goes through four stages: stagnation, heat, deficiency, and injury, and each stage can be divided into different TCM syndromes. This new system has been adopted by the *International Traditional Chinese Medicine Guideline for Diagnostic and Treatment Principles of Diabetes* (Lian et al. 2020), and when compared with the traditional clinical diagnosis and treatment mode of TCM, it is more conducive to establishing a comprehensive understanding of the law of disease development and evolution (Song et al. 2018) (Fig. 1, Table S1).

But how can we clarify the scientific connotation and clinical advantages of the new system? It is necessary to deeply understand the macro- and micro-phenotypic characteristics of different types, different stages, and different syndromes. In recent years, relevant research has been published successively, and we will attempt to make a brief summary as follows.

## Phenomics and the Classification of Diabetes

Both Pi Dan and Xiao Dan originate from the *Yellow Emperor's Internal Classic*, describing two different pathological states of diabetes. Specifically, an unhealthy diet and lifestyle over the long term are key factors leading to the formation of Pi Dan. Obesity and insulin resistance are its typical characteristics, and may be accompanied by symptoms, such as facial flushing, constipation, sweet taste in the mouth, and sticky sweat. In terms of laboratory examination indicators, most Pi Dan patients are also accompanied by multiple metabolic disorders, such as blood lipids, blood pressure, and purine levels, which are closely related to the stagnation of phlegm and dampness in TCM. Notably, epidemiological investigation conducted by our team showed that Pi Dan has become the main type among people suffering from type 2 diabetes mellitus (T2DM) (Wei et al. 2010). By comparison, Xiao Dan patients are commonly seen in type 1 diabetes mellitus (T1DM) and non-obese T2DM with poor glycemic control, and gradual weight loss and decreased insulin secretion are significant characteristics of this group, along with symptoms such as fatigue, cloudy urine, and blurred vision, as well as laboratory test findings, such as increased urinary protein, decreased nerve conduction velocity, and retinal hemorrhage. In the view of TCM, Xiao Dan patients are usually closely related to the Qi-Blood Deficiency and Yin Deficiency with Internal Heat (Tong et al. 2009). With the progress of modern medical technology, the micro-phenotypic differences hidden behind the Pi Dan and Xiao Dan are constantly being revealed. For example, results of metabolomics studies suggested that phospholipid metabolites, including choline, glycerophosphoethanolamine, and glycerophosphocholine were potential biomarkers of obesity-related insulin resistance (Al-Sulaiti et al. 2019); proteomic analyses showed that lipocalin, matrix symporter-1, and a range of protein kinases might differ in the pathogenesis of obese and non-obese subtypes of diabetes (Wang et al. 2013); meanwhile, with the increasing glucose metabolic abnormalities and obesity, plasma endotoxin levels tended to increase in subjects (Liu et al. 2013). To more clearly display the phenotype characteristics related to Pi Dan, we listed some typical studies in Table S2. Correspondingly, the diabetic population with a low body mass index (BMI) also had unique metabolic characteristics, highlighted by a lower total insulin secretory response, endogenous glucose production, visceral adipose tissue, and hepatocyte lipids than the normal weight population, while glucose uptake was significantly higher than that in controls (Lontchi-Yimagou et al. 2022). In addition, a series of multi-omic studies on T1DM have also been reported in succession, which provides us with a deeper understanding of the pathological characteristics of the Xiao Dan population (Yi et al. 2018) (Table S3). All in all, the above studies revealed the phenotype characteristics of obese type and thin type diabetes from different perspectives, which provided an important idea for revealing the scientific connotation of Pi Dan and Xiao Dan.

## Phenomics and the Staging of Diabetes

Based on a large number of previous studies, we considered “stagnation, heat, deficiency and injury” as four major stages of the development of diabetes. Stagnation is the core pathogenesis of prediabetes, and overeating, lack of exercise, and long-term emotional distress are all direct causes contributing to this state. In the theory of TCM, stagnation in Qi and Blood can generate the internal heat, resulting in a series of symptoms such as thirst and hunger; meanwhile, internal heat can also damage the Qi and the body fluid, which may lead to the obstruction of blood circulation and the formation of blood stasis, and ultimately promote the occurrence of a variety of vascular complications related to diabetes (Gou et al. 2020), generally speaking, “heat” and “deficiency” often occur in patients with early and middle-late stages of diabetes, and “injury” usually represents the occurrence of diabetes-related complications. In short, “stagnation, heat, deficiency and injury” is the high generalization of the pathological state of diabetes population in different periods, and a series of clinical symptoms hidden behind them has mapped out TCM phenotypic changes in the development of diabetes, but the molecular connotations are still needs to be further revealed. Several clinical observations have suggested there have begun to show significant changes in the gut microbiota in the pre-diabetic period, which highlighted by a decrease in butyrate-producing bacteria such as *Faecalibacterium spp.* And an increase in opportunistic pathogenic bacteria, such as *Escherichia coli*, accompanied by changes in intestinal and serum metabolites (Allin et al. 2018; Wildberg et al. 2019; Zhong et al. 2019; Wu et al. 2020). With the progress of the disease, the disorders of some gut microbiota related metabolites become more serious; for example, studies have confirmed that the increase of circulating trimethylamine oxide level is association with diabetic retinopathy, cerebrovascular disease, and coronary heart disease and other vascular injury-related diseases (Iatcu et al. 2021). In Table S4, we summarized a series of gut microbiota studies on prediabetes, diabetes, and diabetes-related complications, which also reflected the micro-phenotypic changes at different TCM stage to some extent. Meanwhile, we must realize that the gut microbiota can be affected by multiple factors, not just the disease itself. For example, metformin has been shown to play a pharmacological role in ameliorating glucose metabolism by reducing the abundance of *Bacteroides fragilis*, increasing the content of intestinal glycodeoxycholic acid and inhibiting the activation of the intestinal farnesoid-X-receptor signaling pathway (Sun et al. 2018).

In addition, it cannot be ignored that some metabolites also show predictive value for the development of disease and its complications; in other words, changes in their levels may provide a basis for disease staging. For example, the higher level of isoleucine, leucine, valine, tyrosine, and phenylalanine suggested an increased risk of developing diabetes, which also indicated the transition from stagnation to heat (Guasch-Ferré et al. 2016). In a word, all these studies revealed the phenotypic characteristics of the gut microbiota and host metabolism in different stages of diabetes, providing a research idea for us to better understand diabetes staging.

## Phenomics and the Syndrome Differentiation of Diabetes

After classifying and staging of the diabetes, whether the TCM syndrome can be correctly judged determined the treatment effects to a certain extent. “Syndrome differentiation” is a thinking process that involves highly summarizing the current pathogenesis by analyzing and synthesizing various clinical data (Zhao et al. 2021). Modern medicine focuses more on the diseases itself, while TCM syndrome differentiation is a comprehensive approach to identify the overall state of patients. For example, when a patient shows multiple symptoms, such as elevated blood glucose, dyslipidemia, and hypertension, a Western medicine physician may take the treatment of lowering blood glucose, regulating blood lipid and lowering blood pressure, respectively, while traditional Chinese physicians may try to analyze the core pathogenesis of these disorders through syndrome differentiation, and then adopt a series of treatment schemes aiming at the internal causes of metabolic disorders. With the advancement of phenomics research, the biological connotations behind the TCM syndrome are being gradually revealed. It has been found that, compared with other symptoms, diabetic Yin Deficiency syndrome and Internal Heat syndrome were more closely related to blood glucose levels, dyslipidemia, and C-reactive protein; diabetic Blood Stasis syndrome was positively related to low-density lipoprotein cholesterol (LDL-C) and lower limb atherosclerosis, while it was negatively associated with the level of hemoglobin (Zhuo et al. 2022). For elderly diabetic patients, Yin Deficiency, Qi Deficiency and Blood Stasis are the main types of syndromes, and the number of complications and the course of disease are positively correlated with the degree of deficiency (Fan et al. 2022). Furthermore, omics studies have further deepened the understanding of the TCM syndrome. Xu et al. (2012) analyzed plasma samples of healthy population and T2DM patients with different syndrome types using high-performance liquid chromatography, and found that plasma fatty acid profiles of patients with Qi Deficiency, Qi-Yin Deficiency, and Dampness-Heat syndromes were significantly different, which might be biomarkers to distinguish different T2DM syndromes. Wu et al. (2012) used gas chromatography–mass spectrometry (GC–MS) to analyze carbohydrates in urine samples from patients with T2DM and the healthy population, and found that the xylose and C4 sugar 2 were metabolic markers that distinguished the excess syndrome and the deficiency syndrome. Some scholars have revealed the essence of TCM syndrome from the perspective of the gut microbiota and found that some beneficial bacteria such as *Bifidobacteria* and *Lactobacillus* were significantly reduced in individuals with Damp-Heat syndrome, while some opportunistic pathogens such as *Escherichia coli* showed an increasing trend (Ran et al. 2022). Besides, some new medical frontiers are also becoming important tools to interpret the scientific connotation of TCM syndromes, for example, as the important mediators of intercellular communication, exosomes play an important role in studying the interaction among different tissues or organs (McKay et al. 2021; Fernandez-Gonzalez et al. 2021; Taglauer et al. 2022), which has something in common with the holistic view of TCM theory, and more pioneering research on the relationship between exosomes and TCM syndromes needs to be further developed (Mo et al. 2022). In short, as a unique perspective for understanding diseases, the organic integration of TCM syndrome and the modern medical system is an important path to promote the further development of medicine, and phenomics research is revealing more scientific mysteries behind TCM syndrome.

## Phenomics and the Treatment of Diabetes.

An enormous amount of clinical studies have confirmed the therapeutic effects of TCM on the whole cycle of diabetes (Tian et al. 2019), while its multi-pathway and multi-target molecular mechanisms have not yet been studied in depth. With the advancement of phenomics techniques, several studies conducted in recent years have revealed the molecular phenotypic alterations of herbs or their extracts from different perspectives, partially explaining the scientific connotation of the clinical utility of TCM. Table 1 lists some typical studies of TCM for treating diabetes at different periods (Gao et al. 2018; Shao et al. 2020; Zhang et al. 2020a, b; Zhang et al. 2020a, b; Zhang et al. 2020a, b; Li et al. 2021; Su et al. 2021; Wang et al. 2021; Zhao et al. 2022; Yang et al. 2022; Zhang et al. 2022).

**Table 1:** Phenotypic omics study of diabetes at different stages treated using traditional Chinese medicine

Chinese medicine	Model	Disease	Stage	Basic Phenotype	Molecular phenotype	References
Tangning Ziyabitusi Tablet (*Acorns, Frankincense,* *Bletilla striata, Pomegranate flowers, Eucalyptus vulgaris, Galangal, Tianzhu Huang*, etc.)	Wistar Rats	IGR	Stagnation	Body weight and GLU were decreased, and IR was improved	The abundance of *Christensenellaceae_R-7_group*, *norank_f_norank_o_Clostridia_UCG-014*, *UCG-005*, and *Eubacterium_nodatum_group* was increased, and the abundance of *Adlercreutzia* was increased; besides, the Lymph node CD4 + T cell proportions and immune inflammatory factors were decreased	Zhao et al. (2022)
Tang Yiping *(Astragali Radix., Dioscoreae Rhizoma.., Atractylodes Lancea (Thunb.) DC., Bupleuri Radix., RadixPaeoniaeAlba., Coptis chinensis Franch., Alisma plantago-aquatica Linn. Scrophularia ningpoensis Hemsl. Puerariae Lobatae Radix.*)	Wistar rat	IGT	Stagnation	Body weight and 2hPBG were decreased	16 potential therapeutic target proteins were identified, of which 4 were upregulated and 12 were downregulated, including Rbp4, Fam3b, Flot2; besides, the regulated signal transduction pathways included arginine and proline metabolism, glyceride metabolism, glycerophospholipid metabolism, mTOR, Wnt, and insulin signaling pathways	Li et al. (2021)
*Spatholobus suberectus Dunn*	Diet-induced obesity mice	Obesity with IR	Stagnation	Body weight was decreased and IR and hepatic steatosis were improved	The expression of genes related to adipose tissue activation and thermogenesis involving the regulation of key proteins in the MAPK and AMPK pathways was increased; the gut microbiota disorder caused by the obesity was improved, and the number of anti-obesity and anti-diabetes-related bacteria was significantly increased	Zhang et al. (2019a, b)
Qi Jan mixture (*Astragali Radix.*, *Euonymus alatus (Thunb.)Sieb., Coptis chinensis Franch., Puerariae Lobatae Radix*)	Kkay mouse	T2DM	Heat	TC was decreased, and the result of OGTT and IR were improved	Metabolites were significantly altered in the TCM treatment group, including galactose metabolism, valine, leucine and isoleucine degradation metabolism, aminoacyl-tRNA bioanabolism and alanine, aspartate and glutamate metabolism	Gao et al. (2018)
*Scutellaria baicalensis Georgi. and Coptis chinensis Franch*	SD rats	T2DM	Heat	GLU and blood lipids were decreased and IR was improved	The abundance of some potential enteropathogenic bacteria and LPS-producing bacteria, such as Proteobacteria, Enterobacteriaceae, *Enterobacter*, *Escherichia-Shigella*, and *Enterococcus* was decreased, and the abundance of butyrate-producing bacteria, such as Lachnospiraceae and Prevotellaceae was increased	Zhang et al. (2020a, b)
*Radix Sophorae Flavescentis* extract	SD rat	T2DM	Heat	The levels of FBG, TC, TG, LDL-C, GHb and GSP were decreased	The disturbance of gut microbiota in T2DM model was improved, and abnormalities of lipid, amino acid, and carbohydrate metabolism was reversed	Shao et al. (2020)
*Red ginseng*	Rats	T2DM	Deficiency	TC and GLU were decreased, and IR was improved	In the TCM treatment group, 50 biomarkers showed a downward trend, and the regulated pathways involved D-arginine and D-ornithine metabolism, D-glutamine and D-glutamine Acid metabolism, taurine and hypotaurine metabolism, arginine biosynthesis and tryptophan metabolism	Yang et al. (2022)
*Dendrobium candidum*	BKS.Cg-Dock7m + / + Leprdb/Nju mice	T2DM	Deficiency	FBG was decreased	The relative abundance of key bacterial taxa associated with T2D development, including *Akkermansia* and *Parabacteroides* was increased	Wang et al. (2021)
ShenQi compound recipe (*ginseng, milkvetch root, radix snakegourd root, rehmannia root, danshen root, asiatic cornelian cherry fruit, common yam rhizome, rhubarb root, rhizome*)	GK rats	T2DM	Deficiency	Blood glucose variability was decreased, and IR was improved	The relative abundance of *Prevotellaceae*, *Butyricimonas*, *Bacteroides*, *Blautia* and *Roseburia* was increased, and the abundance of *Lactobacillus* and *Rothia* was decreased; besides, the expression of 40 metabolites, involving gluconeogenesis/glycolysis, amino acid metabolism, lipid metabolism, citric acid cycle and butyric acid metabolism was improved	Zhang et al. (2022)
Jin-Mai-Tong Decoction (*dodder seed, glossy privet fruit, yerbadetajo herb, common selfheal fruit-spike, lychee seed, scorpion, cassia bark, yanhusuo, peach seed, cassia seed, manchurian wildginger, leech*)	SD rats	DPN	Injury	The density of intra-epidermal nerve fibers and the density of sub-epidermal nerve fibers was increased	A total of 21 metabolites were identified as potential biomarkers related to the therapeutic effect of Jin-Mai-Tong Decoction, and these metabolites were mainly involved in lipid metabolism, tricarboxylic acid cycle, amino acid metabolism, etc	Zhang et al. (2020a, b)
San-Huang-Yi-Shen Capsule (*milkvetch root, American ginseng, asiatic cornelian cherry fruit, dodder seed, solomonseal rhizome, rehmannia root, gordon euryale seed, motherwort herb, danshen root, sichuan lovage rhizome, largehead atractylodes rhizome, debark peony root*)	SD rats	DKD	Injury	Body weight and GLU were decreased, and the urine protein and renal pathological damage were improved	A series of gut microbiota and serum metabolites were regulated, and *Lactobacillus*, *Candidatus_Saccharimonas*, *Ruminococcaceae UCG-005*, *anaerobic bacteria*, *Bacteroides* and *Christensenellaceae_R-7_group* were closely related to most of the physiological data and differential metabolites after San-Huang-Yi-Shen Capsule treatment	Su et al. (2021)

AMPK, AMP-activated protein kinase; DPN, diabetes peripheral neuropathy; FAM3B, pancreatic-derived factor (also named as PANDER); FBG, fasting blood glucose; FLO-2, flotillin-2; GLU, glucose; HDL-c, high-density lipoprotein cholesterol; IGR, impaired glucose regulation; IGT, impaired glucose tolerance; IL-6, interleukin-6; IR, insulin resistance; LPS, lipopolysaccharide; mTOR, mammalian target of rapamycin; MAPK, mitogen-activated protein kinases; MyD88, myeloid differentiation factor 88; NF-κB, nuclear factor of κB; PBG, postprandial blood glucose; Rbp4, retinol-binding protein; T2DM, type 2 diabetes mellitus; TC, total cholesterol; TCM, traditional Chinese medicine; TG, triglyceride; TLR-4, Toll-like receptor-4; TNF-α, tumor necrosis factor-α; Wnt, ZO-1, zonulin-1

## Phenomics and the Inheritance and Innovation of TCM

### Application of Phenomics to Explore the Secret of the Black Box of TCM

Medical concepts, such as holism, individuation, and preventive treatment of disease, run through all aspects of TCM diagnosis and treatment, and how to reveal the scientific connotation is the key to maintaining the inheritance of TCM. Phenomics is the combination of multi-dimensional massive data, which can reflect the current functional status in a more multivariate, comprehensive, and accurate visual perspective. At the same time, when combined with long-term follow-up, the longitudinal change of phenotypic data will also help deepen TCM’s understanding of the rule of disease development. For example, Fire-Heat is a common pathological state in the early stage of diabetes, and clinicians can diagnose it according to a series of clinical symptoms, but what is its essence? Wang et al. (2016) believed that Excessive Fire is mostly related to the release of inflammatory factors, while Deficient Fire can be caused by abnormal regulation of the endocrine axis. Liang (2019) believed that the neuro-endocrine-immune system function was in an overactive state in most patients with Heat syndrome, which would lead to a significantly enhanced and accelerated response to the immune stimulation. Li et al. (2019) used the network pharmacology method to reveal that Internal Fire might be related to the oxidative/nitrative stress, inflammatory response, and infection. Taking the diabetes population as the research object, Zhou (2013) carried out the correlation analysis between syndrome elements and clinical indicators, and found that glycolipid metabolic disorder and pancreatic islet function damage were the core elements that led to the Heat pathogenesis. In addition, some scholars also conducted relevant exploratory studies on the Heat state of T2DM from the perspective of chronic low-grade inflammation (Pang et al. 2011). In the clinical practice of TCM, constitutional characteristics have always been the focus of attention. At present, phenomics research on different constitutions is carried out rapidly, and the hidden biological codes behind constitutions are gradually being unraveled (Huang et al. 2019). Juho Shin and colleagues found that obese individuals with phlegm–dampness constitution had higher BMI, waist circumference, hip circumference, and an altered composition of their gut microbiota, such as the decreased levels of *Faecalibacterium* (Shin et al. 2022).

### Applying of Phenomics to Build a Bridge Between TCM and Western Medicine

The differences in the medical ideas of TCM and Western medicine lead to the different emphasis on clinical phenotypes, and the complementary advantage and cross-integration are inevitable directions of the future medical development. Using phenomic research methods to deeply understand the thinking mode of TCM and Western medicine is of great significance for medical innovation. First, phenomics is an innovative research strategy to clarify the essence of TCM theory and the mechanism of curative effects. As mentioned above, phenomics can comprehensively and systematically analyze a clinical phenomenon from a multi-dimensional perspective, establish internal logic through scientific data, and profoundly answer the questions about “what” and “why”. For example, for a long time, the understanding of the spleen in TCM has mostly stayed at the level of functional state, and it is believed that the dysfunction of the spleen is closely related to the occurrence and development of diabetes. With the deepening of research, scholars gradually realized that the function of the spleen governing transportation and transformation not only involved the digestion and absorption of the gastrointestinal tract, but also included the gut microbiota and its complex interaction with the liver, gallbladder, pancreas, and other organs. It also reminds us that the overall health benefits brought by coordinating the spleen and stomach need to be comprehensively evaluated from the multi-dimensional phenotype. On the one hand, we should evaluate direct symptoms caused by spleen and stomach dysfunction, such as loose stools, constipation, stomachache, bloating, burping, and pantothenic acid; on the other hand, we should also pay attention to a series of health problems related to the spleen and stomach, including but not limited to fatigue, depression, disorders of glucose and lipid metabolism, decreased immunity, etc., at the same time, the changes of the gut microbiota, circulating metabolites, and other microscopic signs should also be evaluated (Zhang et al. 2019a, b). Second, clinical phenotype from the characteristic perspective of TCM has the enlightenment and reference significance for the development of modern medicine. For example, the observation of the tongue is a unique procedure in TCM diagnosis, and with the development of image processing, artificial intelligence, and other technologies, the research on tongue manifestation is moving toward objectification, standardization, and precision. In recent years, some progress has been made in studies of the correlation between tongue images and clinical indicators, such as predicting the development of diabetes based on tongue images (Jiang and Xu 2019; Sun et al. 2021). Through a prospective study, Guan et al. (2020) found that cyanotic tongue, yellow fur, greasy fur, cracked tongue, and teeth-printed tongue were all positively related factors to the occurrence of diabetic foot, and the incidence rate of diabetic foot when purple tongue appeared was 12.53 times that of red tongue. Changes in the color and shape of the sublingual vessel also have important reference value for the early warning of diabetic complications. A series of studies have shown that there were varying degrees of thickening, lengthening, tortuosity, and static blood of the sublingual vessel in patients with diabetic vascular disease, which was of great significance for understanding the dynamic changes of the disease (Jiang and Xu 2019). Generally speaking, a thick and greasy tongue coating indicates a reduced transportation and transformation ability of spleen and stomach, and in TCM theory, this phenotype suggested the existence of a higher risk of suffering from glycolipid metabolism disorders, but more in-depth mechanism exploration is still lacking. Our team previously performed the metagenomic sequencing analysis on the yellow thick greasy tongue coating of T2DM and healthy people, and found that *Actinobacteria*, *Leptotrichia*, and *Leptotrichia* might be bacterial biomarkers of tongue coating in T2DM (Liu 2020). In short, phenomics, as a universal language, enhances the communication and dialog between TCM and modern medicine, and lays the foundation for the medical innovation.

### “State-Target theory” and the Future Development of TCM Phenomics

At present, although remarkable achievements have been made in TCM phenomics research, there are still a lot of challenges to be solved, which are highlighted in the following points. First, due to the lack of standardized evaluation criteria for TCM syndrome, modern research on the nature of TCM syndrome is still in the exploratory stage. Second, most of the TCM phenomics research only reflects the current data characteristics, and there is a general lack of phenotypic change data based on the long-term follow-up. Additionally, how can we apply TCM phenomics research results to promote clinical efficacy and new drug development? The research path remains to be clarified.

**Fig. 2:**
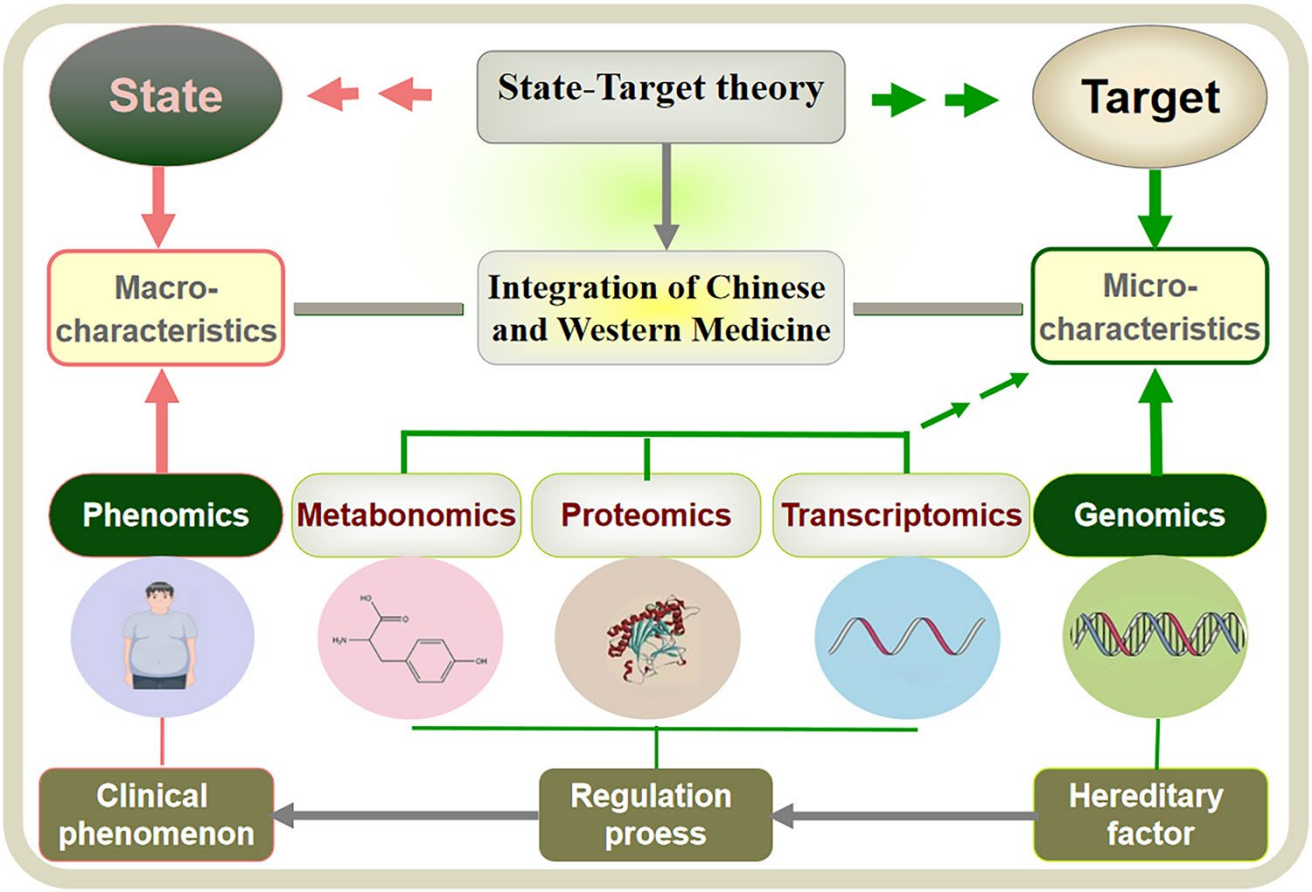
“State-Target theory” and the TCM phenomics

Based on modern clinical characteristics and advantages of TCM, our team creatively proposed the concept of “State-Target theory (STT) (Tong 2021)”. “State” mainly includes three meanings: the current syndrome, the rule of disease development, and the prognosis of disease, and to adjust the state means correcting the imbalance of the human body, thereby reconciling the state of Yin and Yang and inhibiting the progression of the disease. As the characteristic and advantage of TCM, adjusting the state fully reflects the holistic thinking of TCM, and can solve the dilemma that chemical drugs mainly acting on a single target are difficult to treat multiple metabolic disorders. “Target” mainly includes some key focuses in diseases, symptoms, and examination indices, and after thousands of years of development, TCM doctors have accumulated rich experience in improving clinical symptoms, but due to the limitations of the times, ancient doctors usually do not have a deep understanding of the disease mechanism, so that there are significant disadvantages in the application of herbal medicine to improve clinical indicators. In short, state represents a whole healthy network, and targets are key nodes in the network, and conducting the phenomics research from two main lines of “state” and “target” can comprehensively and accurately clarify the therapeutic mechanism of TCM. Still taking diabetes as an example, Danxi Zhu (TCM Scholar of the Yuan Dynasty) first proposed a new syndrome differentiation system (Three-Xiao syndrome differentiation) according to positions of the main syndrome involved in diabetes, and initially described the dynamic evolution rule of the whole process of disease development, at the same time, Danxi Zhu also paid a special attention to the main symptoms of different stages of diabetes, which was also the key targets of the treatment, thus establishing the preliminary framework of “state” and “target” in the treatment of diabetes (He et al. 2020). Today, phenomics research under the coordination of STT has achieved leapfrog development in two aspects. First, by learning from the scientific understanding of the pathophysiology and development law of “diseases” in modern medicine, TCM's understanding on a certain kind of disease has broken through the category of syndromes, and replaced it with a systematic cognition containing classification-staging-syndrome, so as to help fully grasp the trend of disease progression. Second, with the development of modern technology, people's understanding on disease phenotypes has gone far beyond the range of the naked eye, which extends the category of phenotypes from clinical phenomena to molecular level, helping to further deepen the research of the “state” and the “target” of TCM. In a word, the research idea of STT can make the research of TCM phenotype move toward standardization, precision, and practicality (Fig. 2).

## Conclusion

The application of phenomics to carry out TCM research fully reflects the thinking mode of “inheritance and innovation”, providing ideas for deepening the temporal and spatial evolution of Diseases–Syndromes–Symptoms in TCM and is an important tool for exploring the secret of black box. At the same time, the characteristic phenotypes of TCM also provide innovative ideas for modern medicine, which is conducive to the complementarity and integration of TCM and Western medicine.

The modern TCM diagnosis and treatment system provides TCM scheme for clinical treatment of diabetes, and it is urgent to carry out standardized TCM phenomics researches on diabetes, so as to further clarify the scientific connotation of TCM classification, staging, syndrome differentiation, and treatment. Facing the research predicament of TCM phenomics, STT provides a clear research path: on one hand, further clarify the scientific connotation of classification, staging, and syndrome differentiation from the overall perspective; on the other hand, focus on analyzing the key targets of TCM in the disease system regulation network, and finally, clarify the therapeutic mechanism, and greatly promote the process of modernization of TCM and the integration of Chinese and Western medicine.

## Supplementary Information

Below is the link to the electronic supplementary material.

Supplementary file1 (DOC 100 KB)

## Data Availability

This review does not involve the statistical analysis of research data.

## Abbreviations

BMI: Body mass index
TCM: Traditional Chinese medicine
T2DM: Type 2 diabetes mellitus
T1DM: Type 1 diabetes mellitus
LDL-C: Low-density lipoprotein cholesterol
GC–MS: Gas chromatography–mass spectrometry
STT: State-target theory
