# Supplementary material for: Investigation on Phenomics of Traditional Chinese Medicine from the Diabetes
Source: Phenomics. 2024 Sep 2;4(3):257–68. doi: 10.1007/s43657-023-00146-6 (PMC11467137; doi:10.1007/s43657-023-00146-6)
Supplement: Supplementary file 1 — Supplementary file1 (DOC 100 KB) [file 43657_2023_146_MOESM1_ESM.doc]

**Supplementary Materials for**

**Investigation on Phenomics of Traditional Chinese Medicine from the Diabetes**

| **Table S1. “Classification, staging and syndrome differentiation strategy” of diabetes and the main clinical macro-phenotype** | | | |
| --- | --- | --- | --- |
| **classification** | **staging** | **syndrome differentiation** | **Typical clinical macro-phenotype** |
| Pi Dan | Stagnation | Spleen-obstruction and liver-depression | Abdominal distention and fullness, inhibited defecation, depression and nervousness |
|  |  | Spleen and stomach obstruction | Abdominal obesity, abdominal distention and fullness, frequent belching and farting |
|  | Heat | Stagnant heat of liver and stomach | Ruddy complexion, irritable, dry mouth and itter, abdominal distension |
|  |  | Sthenic heat of stomach and intestine | Abdominal fullness and distention, constipation, increased eating with rapid hunger |
|  |  | Intestinal damp heat | Abdominal distention, sticky and foul stool, yellow urine, halitosis |
|  |  | Excessive noxious heat | Thirsty and fondness for cold drink, boredom, dry skin or sores |
|  |  | Intermingled phlegm-heat | Fat, swollen abdomen, dry mouth and thirst, abdominal distension |
|  | Deficiency | Spleen-deficiency and stomach-stagnation | Stomach fullness, fatigue and anorexia, bitterness, loose stool |
|  |  | Deficiency-cold of spleen and stomach | Afraid of cold, stomach distension and stomachache, fatigue and anorexia, loose stool |
|  |  | Spleen-kidney yang deficiency | Coldness of the waist and knee, frequent night urination, afraid of cold, loose stool |
|  | Injury | Phlegm-stasis stagnating in the meridian | Limb numbness and pain, chest tightness and tingling, dark purple lips and tongue |
|  |  | Blood stasis and toxin damaging collaterals | apoplectic hemiplegia, slurred speech, eyeground hemorrhage |
| Xiao Dan | Stagnation | Liver depression and qi stagnation | Depression and nervousness, dry mouth and thirsty, swelling of lateral thorax |
|  |  | External contraction of heat evil | Fever and aversion to cold, stuffy nose and runny nose, head and body pain |
|  | Heat | Exuberant lung-stomach heat | Extreme thirst, increased eating with rapid hunger, increased water consumption |
|  | Deficiency | Yin deficiency and fire vigor | Dysphoria with a smothery sensation, irritable, mouth dry and thirsty, insomnia |
|  |  | Excessive heat damaging fluid | Extreme thirst, increased water consumption, fondness of cold drink, fatigue |
|  |  | Deficiency of both qi and yin | Fatigue, emaciation, increased water consumption, increased sweating |
|  |  | Yin and yang deficiency | Frequent urination, fatigued spirit and lack of strength, chilly sensation and the cold limbs |
|  |  | Deficiency of liver and kidney yin | Frequent urination, turbid urine, blurred vision, lumbar debility, dysphoria |
|  |  | Spleen-kidney yang deficiency | Coldness of the waist and knee, frequent night urination, afraid of cold, loose stool |
|  | Injury | Blood stasis and toxin damaging collaterals | Apoplectic hemiplegia, slurred speech, eyeground hemorrhage |
|  |  | Impairment of both qi and the body fluid | Extreme exhaustion, dry throat and mouth, withered skin and hair, hectic fever |

| **Table S2. Microphenotypic characteristics in obese type diabetes population (*Pi Dan*)** | | | |
| --- | --- | --- | --- |
| **Subject population** | **Phenotypic category** | **Microphenotypic characteristics** | **Reference** |
| Obese people with T2DM and prediabetes | Circulating biomarkers | With the increase in glucose metabolic abnormalities and BMI, the level of plasma endotoxin was gradually increased. | [1] |
| Obese people with insulin resistance and T2DM | Circulating metabolic biomarkers | Phospholipid metabolites, including choline, glycerophosphoethanolamine and glycerophosphocholine were potential biomarkers of obesity-related insulin resistance. | [2] |
| Obese T2DM | Circulating proteome biomarkers | Adiponectin was down-regulated while stromal interaction molecule 1 was up-regulated in the obese T2DM population. | [3] |
| Obese T2DM | Circulating metabolic biomarkers | Total cholesterol in HDL and cholesterol esters in large VLDL particles  might be an important biomarker in the identification of early development of obesity associated T2DM risk. | [4] |
| T2DM patients with abdominal obesity | Circulating proteome biomarkers | Seven proteins (Alpha-1-antichymotrypsin, Alpha-1-antitrypsin, Apolipoprotein A-I, haptoglobin, retinol-binding protein 4, transthyretin, and zinc-alpha2-glycoprotein) might act as protein biomarkers for diabetes progression in overweight patients. | [5] |
| Obese T2DM | White adipose tissue transcriptome | In obese T2DM patients, the expression amplitudes of core clock genes was reduced, meanwhile, the total number of rhythmic genes was also strongly reduced and the rhythm in metabolic pathways was lost. | [6] |
| Obese T2DM | Gut microbiota | Obesity was associated with notable changes in *Akkermansia*, *Faecalibacterium*, *Oscillibacter*, and *Alistipes* and T2D was related with nominal increases in *Escherichia/Shigella*. | [7] |

| **Table S3. Microphenotypic characteristics in thin type diabetes population (*Xiao Dan*)** | | | |
| --- | --- | --- | --- |
| **Subject population** | **Phenotypic category** | **Microphenotypic characteristics** | **Reference** |
| Children with new-onset T1D | Gut microbiota | *Bacteroides* functions related to the immune response was up-regulated；  SCFA producing bacteria *F. prausnitzii* and *Bifidobacterium* activity were down-regulated. | [8] |
| Children with new-onset T1D | Gut microbiota and their metabolites | LPS biosynthesis was increased and butyrate production and bile acid metabolism was decreased;the levels of five metabolites (L-pyroglutamic acid, pterine, 5-hydroxytryptophol, N1-acetylspermine, and 3-(3-hydroxyphenyl)-3-hydroxypropanoic acid) increased significantly, while those of 21 metabolites, including glycoursodeoxycholic acid, glycochenodeoxycholic acid, and DLbenzylsuccinic acid, decreased significantly. | [9] |
| Patients with latent autoimmune diabetes | Gut microbiota | SCFA producing bacteria *Faecalibacterium*, *Roseburia* and *Blautia* were significantly decreased. | [10] |
| T1D related kidney disease | Urinary metabolites | Seven urinary metabolites were associated with overall progression: leucine, valine,isoleucine,pseudouridine,threonine, citrate, 2-Hydroxyisobutyrate. | [11] |
| T1D patients | Pancreatic tissue proteomics | KEGG pathway and functional network analyses of the DEPs reveal dysregulations to pancreatic exocrine function, complement coagulation cascades, and extracellular matrix receptor interaction pathways in T1D. | [12] |
| T1D patients | Transcriptome of peripheral blood mononuclear cells | Seven hub genes (DDIT4, ESCO2, SH3BP4, PRICKLE1, EPM2AIP1, KCNJ15 and GRM8) could well predict the occurrence of T1DM, and these hub genes may be mainly involved in the changes of biological functions such as inflammation, infection, immunity, cancer, and apoptosis. | [13] |

| **Table S4. Changes of the gut microbiota or their metabolites in people with diabetes at different stages** | | | |
| --- | --- | --- | --- |
| **Subject population** | **Staging of diabetes** | **Characteristics of the gut microbiota** | **Reference** |
| Adults with prediabetes, overweight, insulin resistance, dyslipidaemia and low-grade inflammation | Stagnation | *Clostridium* and *Akkermansia muciniphila* showed a significant downward trend. | [14] |
| Population with high risk of diabetes, impaired fasting blood glucose and impaired glucose tolerance | Stagnation | Some butyrate producing bacteria, such as *Faecalibacterium spp.*, *Clostridium spp.*, *Alistipes spp.*, *Pseudoflavonifractor spp.*,and *Oscillibacter spp.* showed a downward trend. | [15] |
| Prediabetics and newly diagnosed T2DM | Stagnation- Heat | 1. In prediabetics group, *Escherichia coli*, *Streptococcus salivarius* and *Eggerthella spp* increased significantly; 2. In newly diagnosed T2DM group, the abundance of the *Akkermansia muciniphila* and *Clostridium bartlettiii* was lower, and the abundance of the *Bacteroides caccae*, *Bacteroides finegoldii*, and *Collinella integralis* was higher. | [16] |
| Prediabetics and newly diagnosed T2DM | Stagnation-Heat | 1. In prediabetics group, butyrate-producing bacteria (e.g. *Akkermansia muciniphila*, and *Faecalibacterium prausnitzii*) showed a lower abundance;  2. In newly diagnosed T2DM group, the abundance of *Bacteroides* and *Verrucomicrobiae* was significantly reduced. | [17] |
| Newly diagnosed T2DM | Heat | The aboundance of *Lactobacillus* was significantly higher, whereas *Clostridium coccoides* and *Clostridium leptum* were significantly lower. | [18] |
| Hospitalized patients with T2DM (with or without diabetes related complications) | Deficiency- Injure | The abundance of Proteobacteria and the ratio of Firmicutes/Bacteroidetes were higher, and the short chain fatty acids, bile acids and lipids were significantly disordered. | [19] |
| T2DM and patients with diabetic nephropathy | Deficiency- Injure | The relative abundances of butyrate-producing bacteria (*Clostridium*, *Eubacterium*, and *Roseburia intestinalis*) and potential probiotics (*Lachnospira* and *Intestinibacter*) were significantly reduced in T2DM and DN patients; *Bacteroides stercoris* was significantly enriched in fecal samples from patients with diabetic nephropathy. | [20] |
| Patients with diabetic retinopathy | Injure | Plasma levels of trimethylamine-N-oxide were significantly higher. | [21] |

**Reference**

1. Liu Y, ZhaoT, Hou L (2013) Change and Correlated Factors of Fasting Level of the Plasma Endotoxin in Subjects with Different Glucose Tolerances and Body Mass Indices. J Sichuan Univ(Med Sci Edi) (5):6.
2. Al-Sulaiti H, Diboun I, Agha MV, Mohamed FFS, Atkin S, Dömling AS, Elrayess MA, Mazloum NA (2019) Metabolic signature of obesity-associated insulin resistance and type 2 diabetes. J Transl Med 17(1):348.
3. Wang J, Yu W, Xu J, Feng L, Yang H, Liu X (2013) Plasma proteomic research on obesity subtype and non-obesity subtype of T2DM. Journal of Hygiene Research 42(02):173-178.
4. Ali MK, Kadir MM, Gujral UP, Fatima SS, Iqbal R, Sun YV, Narayan KMV, Ahmad S (2022) Obesity-associated metabolites in relation to type 2 diabetes risk: A prospective nested case-control study of the CARRS cohort. Diabetes Obes Metab 24(10):2008-2016.
5. Kim SW, Choi JW, Yun JW, Chung IS, Cho HC, Song SE, Im SS, Song DK (2019) Proteomics approach to identify serum biomarkers associated with the progression of diabetes in Korean patients with abdominal obesity. PLoS One 14(9):e0222032.
6. Stenvers DJ, Jongejan A, Atiqi S, Vreijling JP, Limonard EJ, Endert E, Baas F, Moerland PD, Fliers E, Kalsbeek A, Bisschop PH (2019) Diurnal rhythms in the white adipose tissue transcriptome are disturbed in obese individuals with type 2 diabetes compared with lean control individuals. Diabetologia 62(4):704-716.
7. Thingholm LB, Rühlemann MC, Koch M, Fuqua B, Laucke G, Boehm R, Bang C, Franzosa EA, Hübenthal M, Rahnavard A, Frost F, Lloyd-Price J, Schirmer M, Lusis AJ, Vulpe CD, Lerch MM, Homuth G, Kacprowski T, Schmidt CO, Nöthlings U, Karlsen TH, Lieb W, Laudes M, Franke A, Huttenhower C (2019) Obese Individuals with and without Type 2 Diabetes Show Different Gut Microbial Functional Capacity and Composition. Cell Host Microbe 26(2):252-264.e10.
8. Levi Mortera S, Marzano V, Vernocchi P, Matteoli MC, Guarrasi V, Gardini S, Del Chierico F, Rapini N, Deodati A, Fierabracci A, Cianfarani S, Putignani L (2022) Functional and Taxonomic Traits of the Gut Microbiota in Type 1 Diabetes Children at the Onset: A Metaproteomic Study. Int J Mol Sci 23(24):15982.
9. Yuan X, Wang R, Han B, Sun C, Chen R, Wei H, Chen L, Du H, Li G, Yang Y, Chen X, Cui L, Xu Z, Fu J, Wu J, Gu W, Chen Z, Fang X, Yang H, Su Z, Wu J, Li Q, Zhang M, Zhou Y, Zhang L, Ji G, Luo F (2022) Functional and metabolic alterations of gut microbiota in children with new-onset type 1 diabetes. Nat Commun 13(1):6356.
10. Fang Y, Zhang C, Shi H, Wei W, Shang J, Zheng R, Yu L, Wang P, Yang J, Deng X, Zhang Y, Tang S, Shi X, Liu Y, Yang H, Yuan Q, Zhai R, Yuan H (2021) Characteristics of the Gut Microbiota and Metabolism in Patients With Latent Autoimmune Diabetes in Adults: A Case-Control Study. Diabetes Care 44(12):2738-2746.
11. Mutter S, Valo E, Aittomäki V, Nybo K, Raivonen L, Thorn LM, Forsblom C, Sandholm N, Würtz P, Groop PH (2022) Urinary metabolite profiling and risk of progression of diabetic nephropathy in 2670 individuals with type 1 diabetes. Diabetologia 65(1):140-149.
12. Woo J, Sudhir PR, Zhang Q (2020) Pancreatic Tissue Proteomics Unveils Key Proteins, Pathways, and Networks Associated with Type 1 Diabetes. Proteomics Clin Appl 14(6):e2000053.
13. Wang Z, Zhang L, Tang F, Yang Z, Wang M, Jia J, Wang D, Yang L, Zhong S, Yuan G (2022) Transcriptome analysis of peripheral blood mononuclear cells in patients with type 1 diabetes mellitus. Endocrine 78(2):270-279.
14. Allin KH, Tremaroli V, Caesar R, Jensen BAH, Damgaard MTF, Bahl MI, Licht TR, Hansen TH, Nielsen T, Dantoft TM, Linneberg A, Jørgensen T, Vestergaard H, Kristiansen K, Franks PW, Hansen T, Bäckhed F, Pedersen O (2018) Aberrant intestinal microbiota in individuals with prediabetes. Diabetologia 61(4):810-820.
15. Wu H, Tremaroli V, Schmidt C, Lundqvist A, Olsson LM, Krämer M, Gummesson A, Perkins R, Bergström G, Bäckhed F (2020) The Gut Microbiota in Prediabetes and Diabetes: A Population-Based Cross-Sectional Study. Cell Metab 32(3):379-390.e373.
16. Zhong H, Ren H, Lu Y, Fang C, Hou G, Yang Z, Chen B, Yang F, Zhao Y, Shi Z, Zhou B, Wu J, Zou H, Zi J, Chen J, Bao X, Hu Y, Gao Y, Zhang J, Xu X, Hou Y, Yang H, Wang J, Liu S, Jia H, Madsen L, Brix S, Kristiansen K, Liu F, Li J (2019) Distinct gut metagenomics and metaproteomics signatures in prediabetics and treatment-naïve type 2 diabetics. EBioMedicine 47:373-383.
17. Zhang X, Shen D, Fang Z, Jie Z, Qiu X, Zhang C, Chen Y, Ji L (2013) Human gut microbiota changes reveal the progression of glucose intolerance. PLoS One 8(8):e71108.
18. Chen PC, Chien YW, Yang SC (2019) The alteration of gut microbiota in newly diagnosed type 2 diabetic patients. Nutrition 63-64:51-56.
19. Zhao L, Lou H, Peng Y, Chen S, Zhang Y, Li X (2019) Comprehensive relationships between gut microbiome and faecal metabolome in individuals with type 2 diabetes and its complications. Endocrine 66(3):526-537.
20. Zhang L, Wang Z, Zhang X, Zhao L, Chu J, Li H, Sun W, Yang C, Wang H, Dai W, Yan S, Chen X, Xu D (2022) Alterations of the Gut Microbiota in Patients with Diabetic Nephropathy. Microbiol Spectr 10(4):e0032422.
21. Liu W, Wang C, Xia Y, Xia W, Liu G, Ren C, Gu Y, Li X, Lu P (2021) Elevated plasma trimethylamine-N-oxide levels are associated with diabetic retinopathy. Acta Diabetol 58(2):221-229.
